# Custom extraction of macular ganglion cell-inner plexiform layer thickness more precisely co-localizes structural measurements with visual fields test grids

**DOI:** 10.1038/s41598-020-75599-0

**Published:** 2020-10-28

**Authors:** Janelle Tong, David Alonso-Caneiro, Nayuta Yoshioka, Michael Kalloniatis, Barbara Zangerl

**Affiliations:** 1 Centre for Eye Health, University of New South Wales, Sydney, NSW 2052 Australia; 2 School of Optometry and Vision Science, University of New South Wales, Sydney, NSW Australia; 3 Contact Lens and Visual Optics Laboratory, Queensland University of Technology, Brisbane, QLD Australia

**Keywords:** Retina, Eye manifestations

## Abstract

We aimed to evaluate methods of extracting optical coherence tomography (OCT)-derived macular ganglion cell-inner plexiform layer (GCIPL) thickness measurements over retinal locations corresponding to standard visual field (VF) test grids. A custom algorithm was developed to automatically extract GCIPL thickness measurements from locations corresponding to Humphrey Field Analyser 10-2 and 30-2 test grids over Goldmann II, III and V stimulus sizes from a healthy cohort of 478 participants. Differences between GCIPL thickness measurements based on VF test grids (VF-based paradigms) and the 8 × 8 grid, as per instrument review software, were analyzed, as were impacts of fovea to optic disc tilt and areas over which GCIPL thickness measurements were extracted. Significant differences between the VF-based paradigms and the 8 × 8 grid were observed at up to 55% of locations across the macula, with the greatest deviations at the fovea (median 25.5 μm, 95% CI 25.24–25.72 μm, *P* < .0001). While significant correlations with fovea to optic disc tilt were noted at up to 33% of locations distributed 6°–8° from the foveal center, there were no marked differences in GCIPL thickness measurements between VF-based paradigms using different stimulus sizes. As such, standard high-density OCT measurement paradigms do not adequately reflect GCIPL measurements at retinal locations tested with standard VF patterns, with the central macular region contributing most to the observed differences and with further correction required for fovea to optic disc tilt. Spatial direction of GCIPL thickness measurements will improve future comparisons of structure and function, thereby improving methods designed to detect pathology affecting the inner retina.

## Introduction

Glaucoma is a progressive optic neuropathy exhibiting selective loss of retinal ganglion cells (GCs), and is a major contributor to significant visual morbidity in developed nations^1, 2^. A hallmark characteristic of glaucoma is concordance between structural and functional damage; that is, defects of the optic nerve head, retinal nerve fiber layer (RNFL) and inner retina mirror associated deficits in visual field (VF) sensitivity^3, 4^. In contrast, structural evidence of glaucoma in the absence of notable functional deficits or vice versa^5^, termed structure–function discordance, introduces a level of diagnostic ambiguity^5^. As such, there is increased interest in understanding the structure–function relationship in detail, with the intention of facilitating earlier detection of glaucoma.

The ability of optical coherence tomography (OCT) to acquire in vivo*,* quantitative RNFL and inner retinal thickness measurements has contributed to its ongoing use in investigations of the structure–function relationship^5^. The macula is a particular region of interest due to its high density of retinal GCs^6^, and given the association between macular inner retinal thickness measurements and central VF loss^7, 8^, early detection of glaucomatous loss at the macula is paramount. As such, several studies have capitalized on the ability of commercial OCT software to obtain macular inner retinal thickness measurements to describe the macular structure–function relationship^9–12^. While using measurement paradigms from commercially available OCT software has the advantage of direct applicability to clinical settings, areas over which measurements are averaged are semi-arbitrary and do not directly coincide with retinal locations stimulated by test targets as per standard VF patterns. In particular, averaged foveal inner retinal measurements commonly include the GC-poor foveal pit, and as a result do not adequately reflect the GC-rich locations directly adjacent to the foveal pit^6, 13^ that constitute the primary retinal loci contributing to high foveal VF sensitivities^14, 15^.

As region-averaged OCT-derived measurements do not directly reflect the cellular tissues contributing to visual function, the phenomenon of structure–function discordance may be in part due to discrepancies in locations over which measurements are derived. Indeed, several studies have highlighted the necessity of comparing structural and functional measures from corresponding retinal locations^7, 16–18^, and we recently partially accounted for this issue via manual extraction of foveal ganglion cell layer (GCL) thicknesses^19, 20^, with these models demonstrating excellent structure–function concordance (coefficients of determination 0.94–0.98)^19^. Additionally, given that different VF stimulus sizes are often tested in investigations of the structure–function relationship^15, 20–23^, the area over which structural measurements are derived may also be pertinent in limiting discordance. Practically speaking, it is important to consider whether high-density measurement paradigms available on commercial OCT software are sufficient surrogates for structural measurements matching retinal locations stimulated by VF test targets, as are utilized in numerous studies^10, 24, 25^, thereby enabling direct translation of research findings to clinical applications. Alternatively, if systematic differences between these methods exist, additional efforts are necessary to ensure structure and function are precisely matched.

Given the discrepancies in macular OCT measurements used in structure–function investigations, we hypothesized that comparisons between OCT measurements directly corresponding to retinal locations stimulated by VF test patterns and a high-density measurement grid available on OCT review software will yield statistically and clinically significant differences. We tested this hypothesis by developing a customized algorithm enabling automated extraction of inner retinal thickness measurements, with spatial adjustment of measurement locations and areas to match VF test grids, and comparisons between the resultant measurements and those directly extracted from the OCT review software were performed. Utilizing structural measures that more accurately reflect underlying visual function has the potential to improve descriptions of the macular structure–function relationship, and in turn guide technologies detecting early glaucomatous changes with greater precision.

## Methods

### Participant cohort and OCT acquisition

This study adhered to the tenets of the Declaration of Helsinki and ethics approval was granted by the University of New South Wales Australia Human Research Ethics Advisory panel. Data from healthy adult participants (Table 1) were retrospectively extracted from patient files who had previously attended the Centre for Eye Health (Sydney, Australia), where all participants underwent comprehensive examination including slit-lamp biomicroscopy examination, intraocular pressure measurement, dilated fundus examination, OCT imaging of the macula and peripapillary RNFL (Cirrus OCT, Carl Zeiss Meditec, Inc., Dublin, CA, USA and Spectralis OCT, Heidelberg Engineering, Heidelberg, Germany) and 24-2 SITA Standard threshold VF testing (Humphrey Field Analyzer (HFA), Carl Zeiss Meditec, Inc.). Exclusion criteria included spherical equivalent refractive error greater than ± 6.00 diopters (D), astigmatism greater than 3.00D, and presence of optic nerve pathology in either eye or macula pathology in the included eye. Informed consent was obtained for all participants included in this study. Data for 250 participants have been included in previous studies^19, 26^.

**Table 1:** Demographic information of the study cohort.

Demographic variable	Mean ± SD	Range
Age	47.83 ± 16.02	20.13 to 84.91
Spherical equivalent refractive error (D)	− 0.62 ± 1.86	− 6.00 to + 3.75
Fovea to optic disc tilt (°)	6.64 ± 3.33	− 5.60 to 16.40

Demographic variable	OD (%)	OS (%)
Eye included	253 (52.9)	225 (47.1)

Demographic variable	Male (%)	Female (%)
Gender	204 (42.7)	274 (57.3)

*SD* standard deviation, *D* diopters, ° degrees, OD right eye, OS left eye, % percentage.

**Figure 1:**
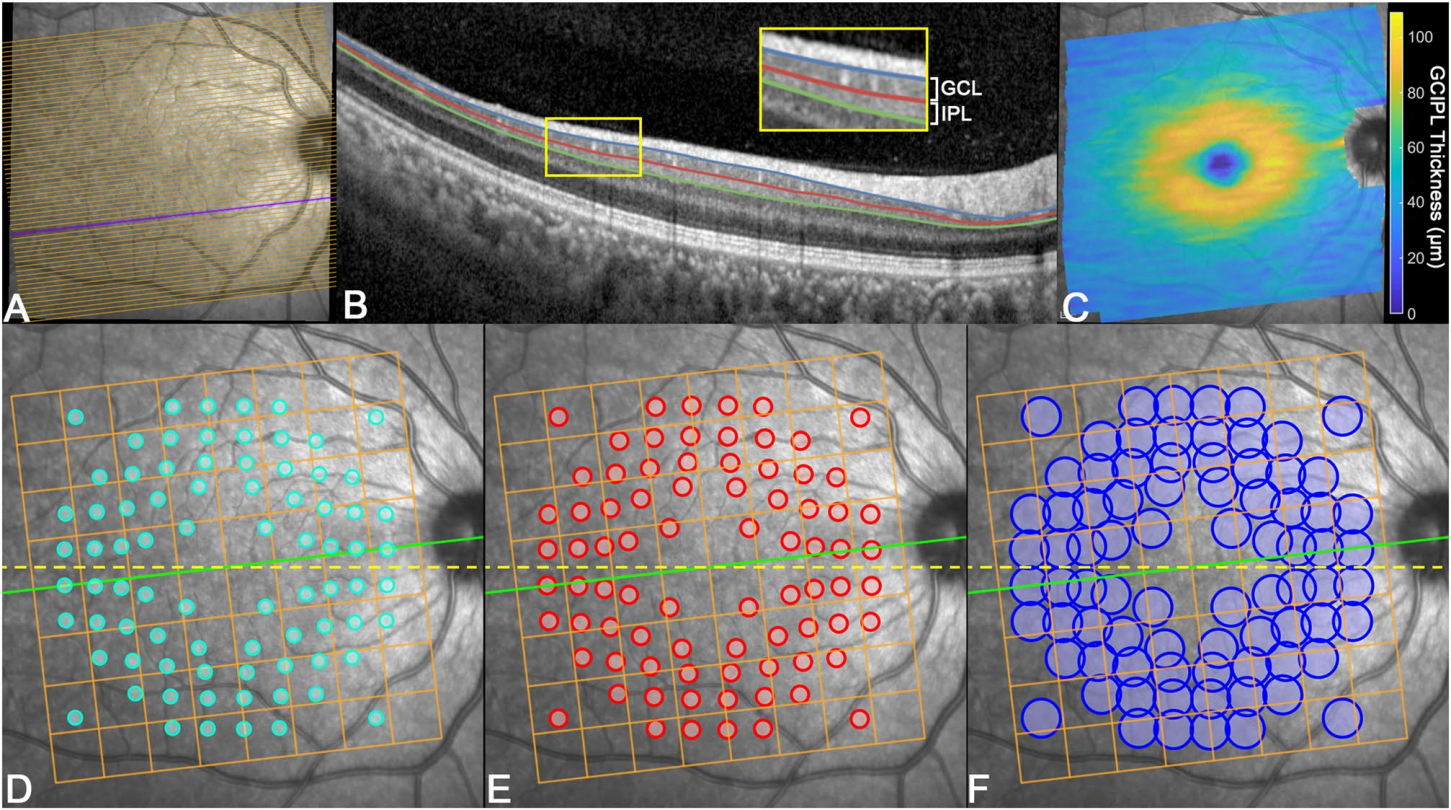
Overview of OCT extraction procedures. (**A**) Spectralis OCT posterior pole volume scans (30° horizontally × 25° vertically), comprised of 61 horizontal OCT images, superimposed on the scanning laser ophthalmoscopy image. (**B**) Segmentation of the 3 boundaries of interest (retinal nerve fiber layer-ganglion cell layer (GCL) boundary [blue line], GCL-inner plexiform layer (IPL) boundary [red line] and IPL-inner nuclear layer boundary [green line]) for a single OCT B-scan highlighted in purple on (**A**). For each B-scan, the boundaries were identified and corresponding thickness measurements for the GCL (between retinal nerve fiber layer-GCL and GCL-IPL boundaries) and IPL (between GCL-IPL and IPL-inner nuclear layer boundaries) were extracted. (**C**) The corresponding ganglion cell-inner plexiform layer (GCIPL) thickness map, calculated by summing colocalized GCL and IPL thicknesses, resampled to the scanning laser ophthalmoscope resolution. (**D**–**F**) Projected locations of Goldmann II (**D**), Goldmann III (**E**) and Goldmann V (**F**) visual field test targets as per the Humphrey Field Analyzer 10-2 test grid with paracentral 30-2 test locations, with correction for lateral displacement of ganglion cells relative to the connecting photoreceptors^16, 20, 27^ and allowances for deviations in fixation secondary to microsaccades (Table 1). The tilt of the 8 × 8 grid, superimposed in orange, was set at the fovea to optic disc tilt (green line) for each participant, while the projected visual field test target grids were set at a tilt of zero (yellow dotted line) regardless of individual fovea to optic disc tilts. This figure was generated using Adobe Photoshop 2020 (Adobe Systems Incorporated, San Jose, CA, USA), with panels A-C generated using Matlab (Mathworks, Natick, MA, USA).

OCT data from one eye per participant were included in this study. Posterior pole OCT was acquired using the Heidelberg Spectralis OCT with the posterior pole volume scan setting. This setting captures the central 30° horizontally by 25° vertically, centered on the foveal pit, with the scanning angle matching the fovea to optic disc tilt as automatically detected using the instrument acquisition module (Fig. 1). Each volume scan consisted of 61 horizontal OCT B-scans spaced 120 μm apart, with each B-scan constructed from averaging a minimum of 9 individual images using the acquisition module’s Automatic Real Time function to reduce noise. A minimum quality score, indicative of the signal strength of the entire volume scan, of 15 decibels (dB) was required for inclusion in further analyses, as per previous inclusion criteria^26^.

OCT data were reviewed using the Heidelberg Eye Explorer review software version 1.10.4.0 (Heidelberg Engineering, Heidelberg, Germany). The 8 × 8 grid, spanning 6880 μm (approximately 24°) horizontally and 6880 μm vertically and comprised of individual grid squares measuring 860 μm horizontally and 860 μm vertically, was selected due to its high sampling density compared with other routinely used paradigms, in addition to its utilization in previous quantitative models of the structure–function relationship^10, 24, 25^. Grid centration and tilt was manually reviewed and adjusted as required to align with the center of the foveal pit and the fovea to optic disc tilt respectively.

The GCL and inner plexiform layer (IPL) were automatically segmented by the instrument review software, with manual review and correction of segmentation as required. Individual grid squares in which segmentation could not be adequately corrected, for example due to shadowing secondary to intraretinal vasculature, impingement of the optic nerve head or poor quality, were excluded as per previous studies^19, 20, 26^. GCL and IPL thickness measurements were directly extracted from the review software, with co-localized GCL and IPL thicknesses summed together to calculate ganglion cell-inner plexiform layer (GCIPL) thickness measurements.

### Algorithm development and data extraction

OCT volume scan data was extracted in RAW file format, with additional data on the location of the foveal center and the angle between the foveal and optic nerve head centers extracted from the corresponding extensible markup language (XML) file. A custom algorithm was developed using Matlab (Mathworks, Natick, MA, USA) and applied to the RAW and XML files for each OCT volume scan. The processes involved in data extraction by this algorithm are summarized in Fig. 1.

Thickness maps spanning the entire acquired area for the GCIPL were generated based on the RAW data, where axial thickness measurements at each pixel location were calculated based on the appropriate boundary positions extracted from the RAW file and an axial resolution of 3.87 μm per pixel (Fig. 1)^28^. To calculate the thickness map size matching the resolution of the scanning laser ophthalmoscope image accompanying the OCT volume scans, the 61 B-scan thickness profiles were interpolated using a bicubic interpolation. The first (superior-most) and last (inferior-most) B-scans were utilized to calculate the dimensions of the standard 8 × 8 grid, matching that from the instrument review software. The 8 × 8 grid was placed over the thickness maps, with grid centration and tilt as specified by the corresponding XML file, and average GCIPL measurements were calculated across each grid square.

Additionally, GCIPL thickness measurements averaged over spatial areas coinciding with projections of Goldmann sizes II, III and V stimuli as per the HFA 10-2 test grid and 12 paracentral locations on the 30-2 test grid (henceforth labelled GII, GIII and GV VF-based paradigms respectively, Fig. 1) were extracted with the algorithm. The rationale behind investigating these test sizes is as follows: GII fulfils complete spatial summation criteria within the macula and has been advocated for use in early glaucomatous detection^14,15,21,22,29^, GIII is the standard test target size used in clinical practice^4^, and GV has demonstrated reduced test–retest variability and has been advocated for use in glaucoma detection and monitoring for progression^23,30^. Projected areas over which measurements were averaged were derived from manufacturer-specified relative sizes of GII, GIII and GV test targets, with allowances for small deviations in fixation secondary to microsaccadic eye movements (Table 2)^31,32^. The locations of the projected VF stimuli were corrected for lateral displacement of GCs relative to the underlying stimulated photoreceptors due to elongation of Henle fibers at the central fovea^16,20^, as per Drasdo et al.’s Fig. 6^27^ and consistent with previous studies applying this methodology^7,16,18,33^. Irrespective of individual fovea to optic disc tilts, the VF-based paradigms were maintained at a tilt of 0° to maintain consistency with natural head positioning during VF testing and therefore ensure that structural measurements were accurately extracted over retinal locations stimulated by VF test points (Fig. 1).

**Table 2:** Effective visual field stimulus sizes projected onto the retina, both as specified by the manufacturer and with allowances for deviations due to microsaccadic eye movements.

	Effective VF stimulus size		Effective VF stimulus size with microsaccade allowance	
	Angle subtended (°)	Area (mm^2^)	Angle subtended (°)	Area (mm^2^)
GII	0.22	0.00298	0.72	0.0433
GIII	0.43	0.0119	0.93	0.0691
GV	1.72	0.191	2.22	0.351

This has been previously reported to be 30 min of arc^31, 32^, equivalent to 0.5°

*VF* visual field, ° degrees, *mm* millimeters, *GII* Goldmann II, *GIII* Goldmann III, *GV* Goldmann V.

### Statistical analysis

Statistical analyses were performed using GraphPad Prism Version 7.04 (GraphPad, La Jolla, CA, USA). All data were converted to right eye format to facilitate direct comparisons between all participants, and normality of GCIPL data was determined using D’Agostino and Pearson normality tests. As the data did not follow a normal distribution, validation of the developed algorithm was performed by applying one sample Wilcoxon tests to analyze overall and pointwise differences between standard and algorithm-derived GCIPL thicknesses across the 8 × 8 grid. GCIPL thickness measurements from each location within each VF-based paradigm were individually compared with those from the corresponding grid square in the 8 × 8 grid with which is shared the greatest area using one sample Wilcoxon tests, with 8 × 8 grid GCIPL measurements extracted directly from the instrument review software. In the circumstances where two VF-based paradigm locations fell within the same grid square in the 8 × 8 grid, both VF-based GCIPL measurements would be compared to the GCIPL measurement from the same grid square. When a location from a VF-based paradigm fell between 2 or more grid squares, comparisons were made with the grid square with which it shared the greatest area, which was determined based on individual fovea to optic disc tilts; further details are available in Supplementary Figure S1. Measurements from a grid square that did not clearly correspond with a location from any VF-based paradigm were excluded from these analyses. To investigate whether greater deviations occur with different fovea to optic disc tilts, Spearman correlations between fovea to optic disc tilts and differences in GCIPL thicknesses were calculated for each location within VF-based paradigms and for all test sizes. Furthermore, rather than assuming linearity of the relationship between fovea to optic disc tilts and differences in GCIPL thicknesses, particularly as inner retinal parameters have been previously demonstrated to show non-linear rates of change^19,26^, extra sums-of-squares F tests were applied to compare the fits of quadratic and linear regression functions, with the better fitting model subsequently used to compare GCIPL thicknesses between 0° and 15° fovea to optic disc tilts. Finally, comparisons of GCIPL at each measurement location between different VF-based paradigms were performed in a pointwise manner using Friedman tests with multiple comparisons. The level of statistical significance was set at *P* < 0.05.

## Results

### Algorithm validation via comparison of 8 × 8 grid measurements

Algorithm validation was performed by comparing GCIPL thickness measurements extracted using the novel algorithm and OCT review software over identical measurement locations. One sample Wilcoxon tests demonstrated that the custom algorithm tended to underestimate GCIPL thicknesses by a median of 0.40 µm (95% CI − 0.50 to − 0.20 μm, *P* < 0.0001). While statistically significant, given that the instrument review software displays thickness measurements rounded to the nearest integer, this small difference can be considered negligible in a clinical setting.

**Figure 2:**
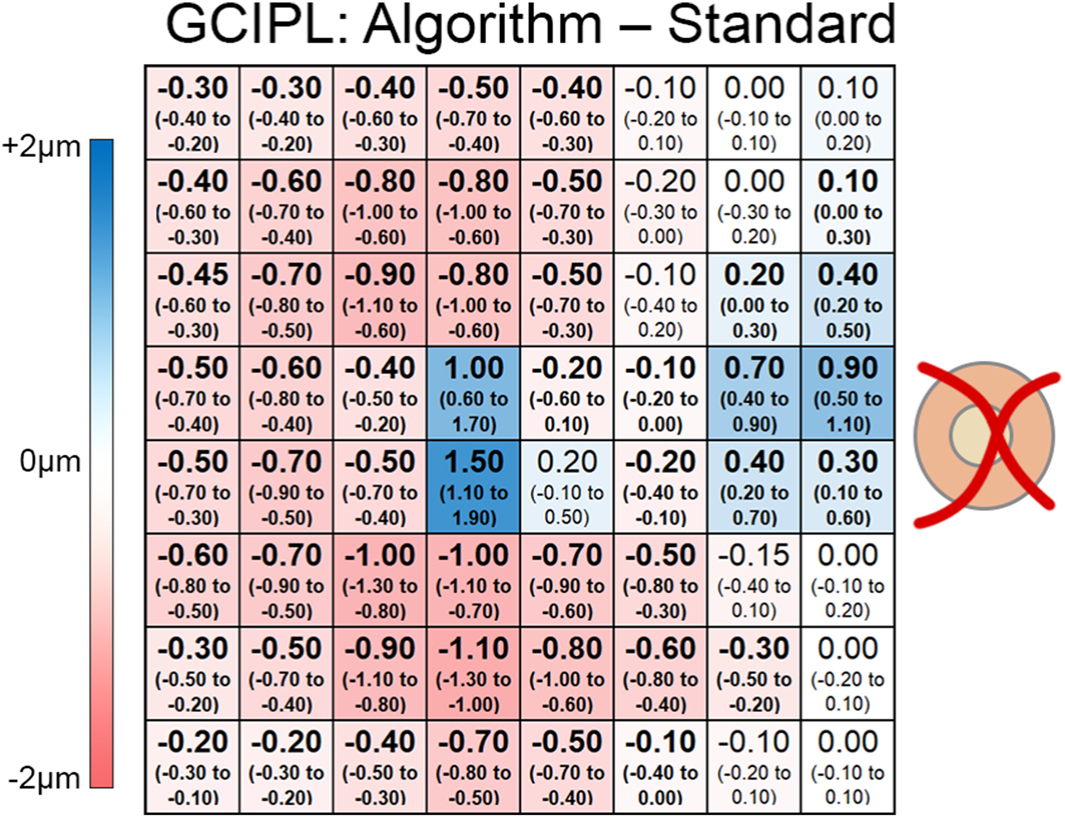
Thickness difference map illustrating deviations in median ganglion cell-inner plexiform layer (GCIPL) thickness measurements calculated via the custom algorithm (algorithm) versus those extracted directly from the instrument review software (standard). Values within each grid square indicate median differences across all participants, and values in brackets indicate the 95% confidence intervals for each location. Values in bold indicate significant deviations (one sample Wilcoxon test, P < 0.05). This figure was generated using Adobe Photoshop 2020 (Adobe Systems Incorporated, San Jose, CA, USA).

The small overall differences between the algorithm and instrument review software can be visualized throughout the 8 × 8 grid when observing differences in individual grid squares (Fig. 2). While most grid squares were flagged as showing significantly reduced thickness measurements when measured with the custom algorithm, these differences did not exceed 1 μm in the majority of locations, with slightly larger differences observed inferotemporal to the foveal center. Meanwhile, the grid squares directly temporal to the grid center and at the nasal macula adjacent to the optic disc showed thicker measurements when extracted using the custom algorithm, although differences were similarly less than 1 μm in most locations. All differences fell well within previously reported coefficients of repeatability for the GCL and IPL^34^, and therefore are within the inter-scan reproducibility limits of the Spectralis OCT.

### VF-based paradigms versus 8 × 8 grid measurements

**Figure 3:**
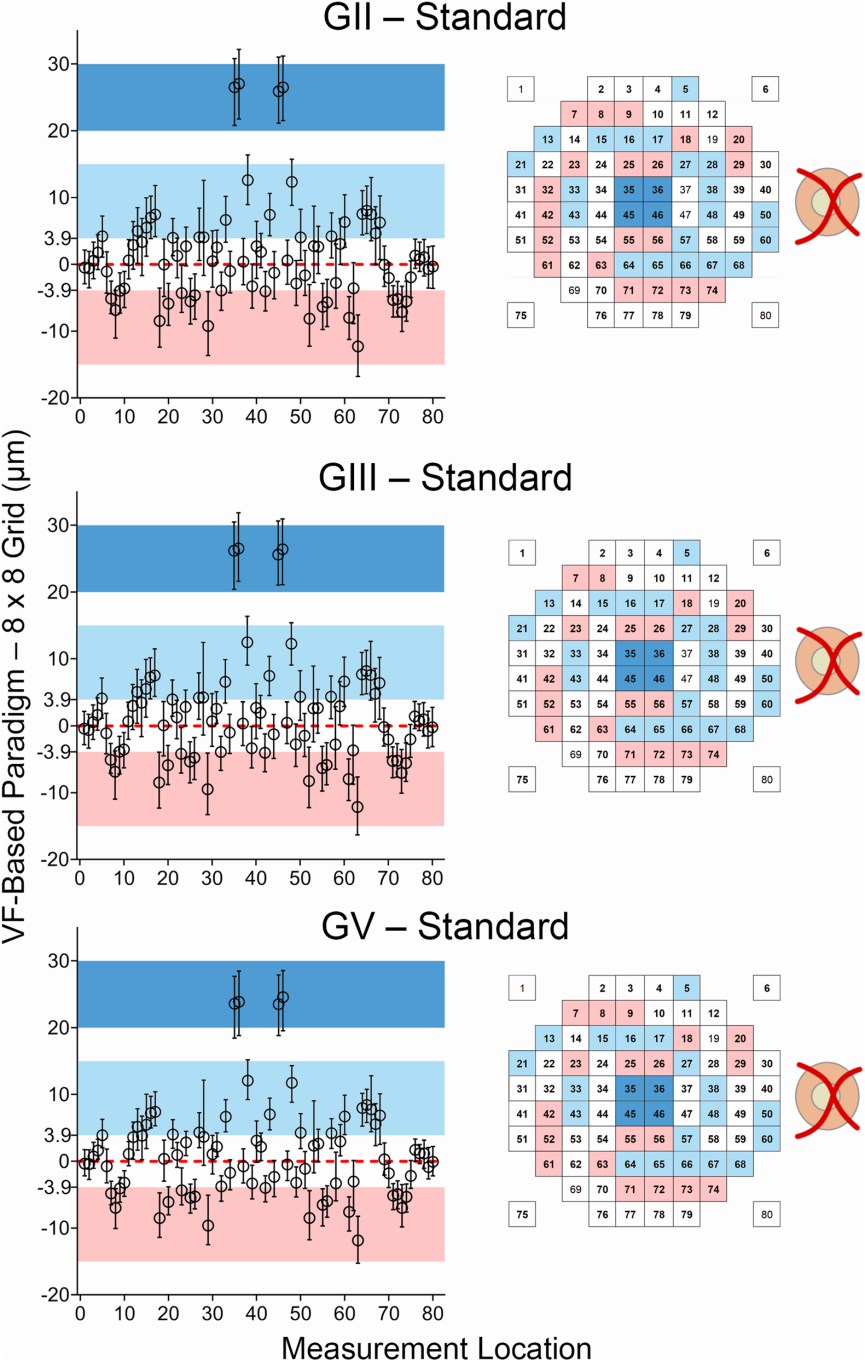
Scatter plots depicting differences in median ganglion cell-inner plexiform layer (GCIPL) thickness measurements as extracted over projected Goldmann II, III and V (GII, GIII and GV) locations as per each visual field (VF)-based paradigm, versus those extracted directly from the instrument review software (standard). Each measurement location as per the VF-based paradigm, labelled as per the figure legend (right column), was compared to the grid square measurement with which it shared the greatest area, as calculated for individual fovea to optic disc tilts. Individual data points (circles) indicate median differences between VF-based paradigms and standard measurements at each location, and error bars indicate interquartile ranges. The red dashed line indicates no difference between the VF-based paradigm and the standard measurements. Individual measurement locations showing the greatest deviation are highlighted in dark blue, corresponding to foveal locations, and pale blue and pale red, corresponding to deviations greater than 3.9 µm and less than − 3.9 µm respectively (i.e. the axial resolution of the Spectralis OCT). Locations in bold in the figure legends indicate significant deviations (one sample Wilcoxon test, P < 0.05). This figure was generated using Adobe Photoshop 2020 (Adobe Systems Incorporated, San Jose, CA, USA), with scatter plots (left column) generated using GraphPad Prism Version 7.04 (GraphPad, La Jolla, CA, USA).

**Figure 4:**
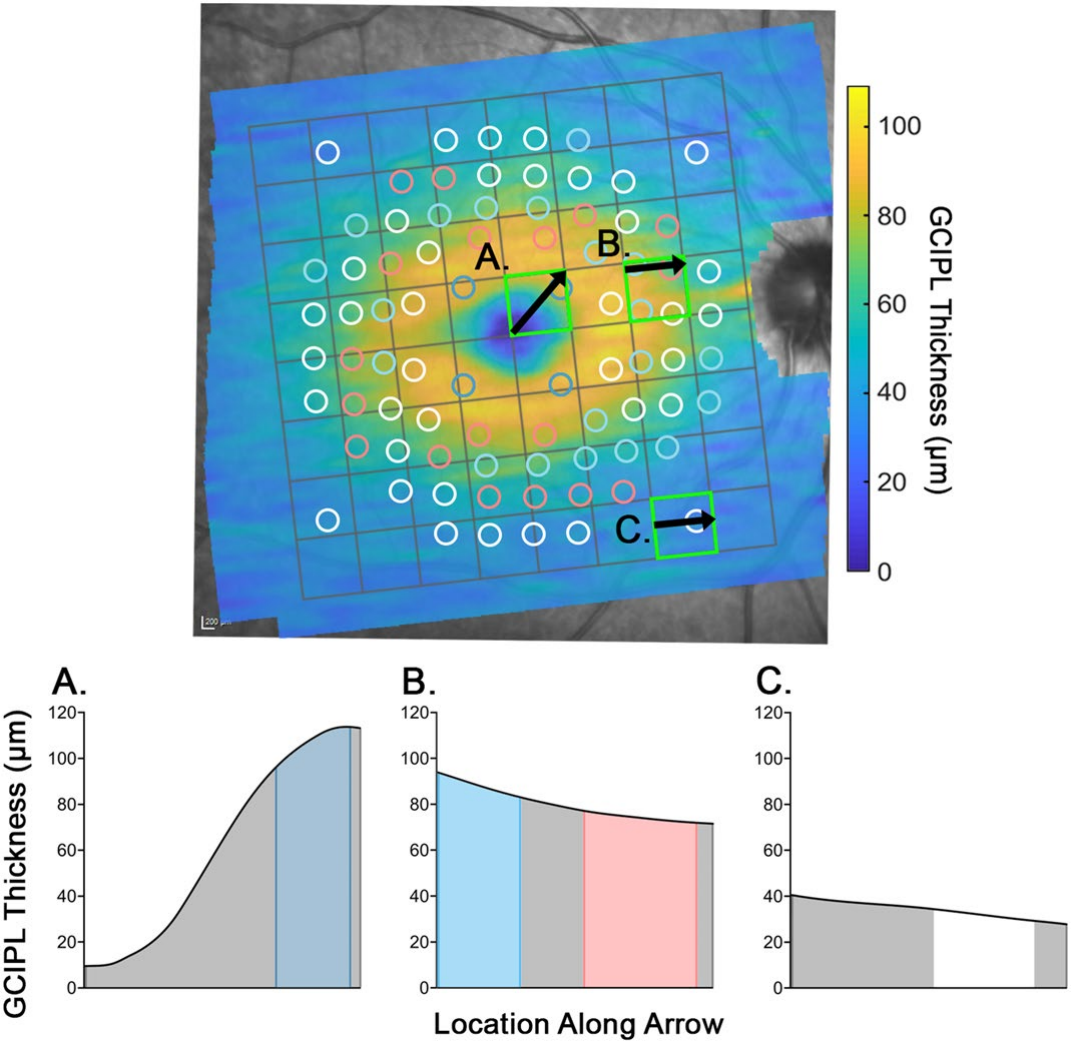
Comparison of ganglion cell-inner plexiform layer (GCIPL) thickness measurements as extracted using the Goldmann III visual field-based (VF-based) paradigm and 8 × 8 grid, with a participant’s GCIPL thickness map underneath. In the top panel, as per Fig. 3 (middle row, right column), the foveal measurements are depicted in dark blue, locations where median differences compared with instrument review software-derived 8 × 8 grid measurements were greater than + 3.9 μm and less than − 3.9 μm are coloured pale blue and pale red respectively, and all other locations are depicted in white. Within the grid squares highlighted in green (**A**–**C**), GCIPL thickness profiles along chosen locations (black arrows) were extracted from the instrument review software (bottom panels (**A**–**C**)). Here, the VF-based paradigm locations falling along each curve are shaded in their respective colours, and grid square locations are shaded in grey. Note that in (**A**) and (**B**), where larger differences between the VF-based paradigm and 8 × 8 grid were observed, GCIPL thickness varies notably within the grid square. Meanwhile, in (**C**), much less variation in GCIPL thickness is observed, resulting in more similar GCIPL thickness measurements between the VF-based paradigm and 8 × 8 grid. This figure was generated using Adobe Photoshop 2020 (Adobe Systems Incorporated, San Jose, CA, USA), with the GCIPL thickness map (top) generated using Matlab (Mathworks, Natick, MA, USA) and the GCIPL thickness profiles (**A**–**C**) generated using GraphPad Prism Version 7.04 (GraphPad, La Jolla, CA, USA).

After validation of the custom algorithm, thickness measurements averaged over locations matching projected locations of HFA test targets and the standard 8 × 8 grid were compared to explore potential systematic differences between the measurement paradigms. Significant differences between thickness measurements from the VF-based paradigms and 8 × 8 grid were observed across the entire macula, regardless of the stimulus size used (Fig. 3), exceeding the differences between the standard and algorithm-derived 8 × 8 grid measurements at most locations. Deviations exceeding 3.9 μm, the axial resolution of the Spectralis OCT, were observed at 41–44 locations (51.25–55% of locations) at the parafovea and central fovea across all VF-based paradigms, corresponding with locations where large variations in GCIPL thickness profiles are typically observed (Fig. 4). At the central four locations directly adjacent to the foveal pit, custom algorithm-derived measurements were a median of 25.50 μm larger than those derived from 8 × 8 grid measurements across all stimulus sizes (95% CI 25.24–25.72 μm, *P* < 0.0001).

**Figure 5:**
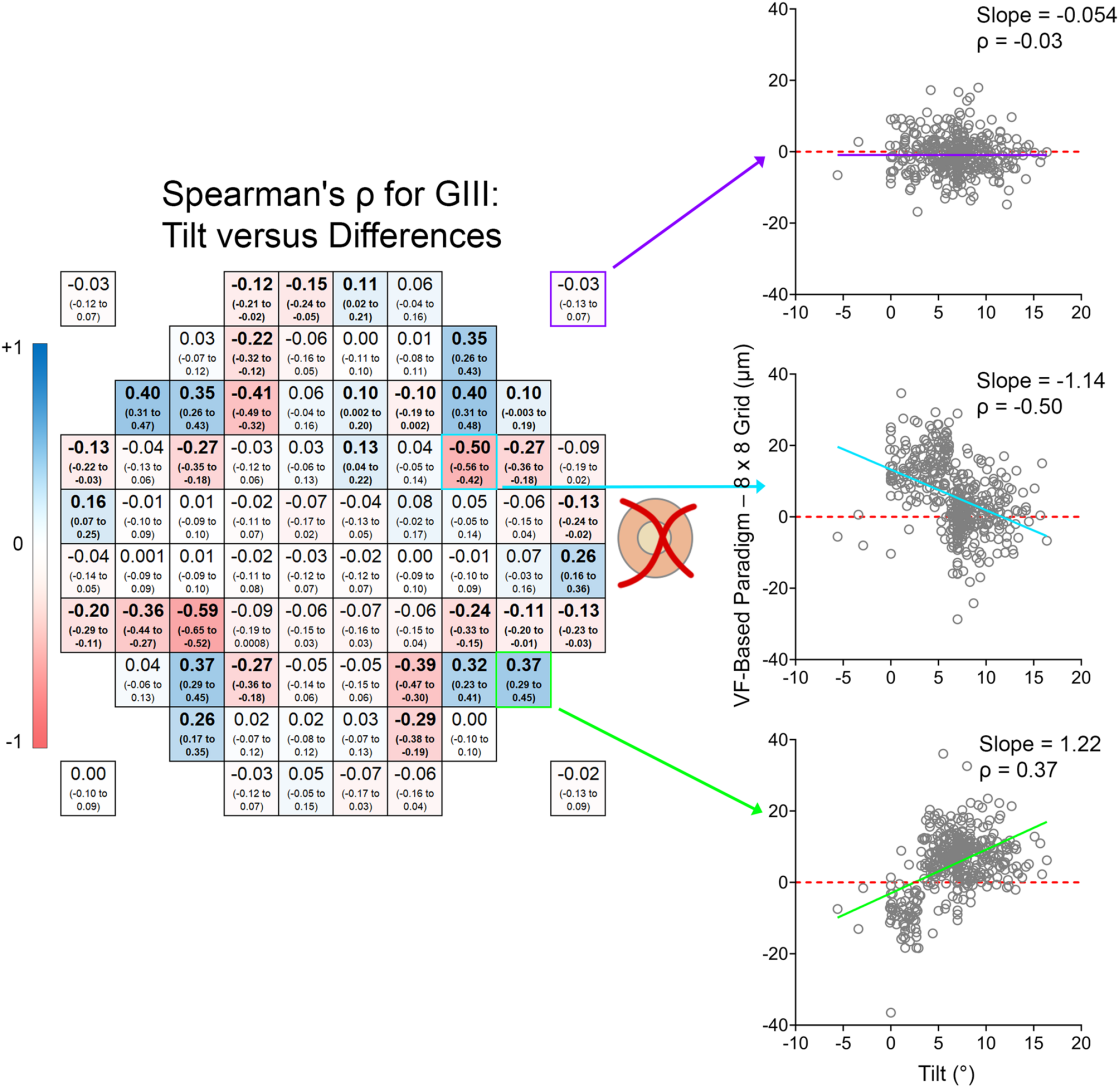
Heat map depicting location-specific variations in Spearman’s rank correlation coefficients (ρ), calculated between fovea to optic disc tilts and differences in ganglion cell-inner plexiform layer (GCIPL) thickness between the Goldmann III (GIII) visual field (VF)-based paradigms and the 8 × 8 grid. Bold values indicate significant correlations (P < 0.05) and values in brackets indicating the 95% confidence intervals of each Spearman’s ρ. For select locations (highlighted in purple, cyan and green), plots showing individual fovea to optic disc tilts against differences in GCIPL thickness and the associated slopes of the linear regressions (purple, cyan and green lines) are included to illustrate how differences between the VF-based paradigms and 8 × 8 grid vary with different fovea to optic disc tilts. The red dashed lines in these plots indicate no correlation between differences in GCIPL thickness measurements and fovea to optic disc tilts. Findings were similar for Goldman II and V VF-based paradigms (available in Supplementary Figure S2). This figure was generated using Adobe Photoshop 2020 (Adobe Systems Incorporated, San Jose, CA, USA), with the scatter plots (right column) generated using GraphPad Prism Version 7.04 (GraphPad, La Jolla, CA, USA).

**Figure 6:**
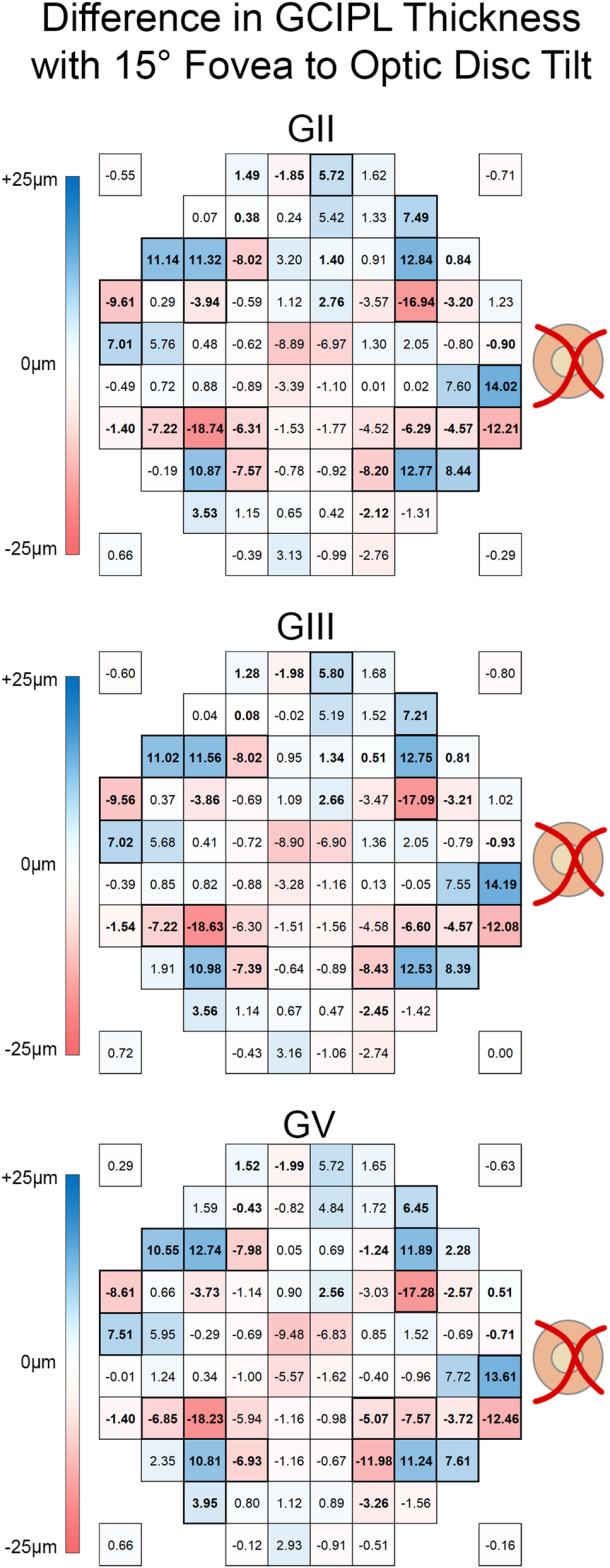
Thickness heat maps depicting differences in ganglion cell-inner plexiform layer (GCIPL) thickness in microns between fovea to optic disc tilts of 0° and 15° for each visual field (VF)-based paradigm. These were calculated from linear and quadratic regression equations describing the relationship between fovea to optic disc tilt and differences in GCIPL thickness between VF-based paradigms and the 8 × 8 grid (Supplementary Figure S4). Bold values indicate significant Spearman correlations (P < 0.05) as per Fig. 5 and Supplementary Figure S4. Locations demarcated by a black outline indicate those that showed significant Spearman correlations and exceeded 3.9 μm. This figure was generated using Adobe Photoshop 2020 (Adobe Systems Incorporated, San Jose, CA, USA).

Differences between VF-based paradigms, kept at 0° tilt to ensure GCIPL measurements were extracted from retinal locations matching VF test grids, and the 8 × 8 grid, positioned according to the fovea to optic disc tilt as per the standard acquisition protocol on Spectralis OCT, may be confounded by interindividual variation in fovea to optic disc tilt. Spearman’s correlations were computed to determine the potential effect of fovea to optic disc tilt, and significant correlations were observed at 30–33 locations (37.5–41.25% of locations), with the largest correlations observed approximately 6°–8° from the foveal center (Fig. 5 and Supplementary Figure S2); these locations also tended to fall on different grid squares with different fovea to optic disc tilts more frequently than locations which did not demonstrate significant correlations (median 85.6% vs. 99.0%, *P* < 0.0001, Supplementary Figure S3). Depending on the location and VF-based paradigm, quadratic and linear regression models variably best described the relationship between fovea to optic disc tilt and difference in GCIPL thickness, however in locations showing the greatest correlations the relationship tended towards a quadratic rather than a linear model (Supplementary Figure S4). Additionally, calculations using the appropriate regression equations demonstrated that with a 15° difference in fovea to optic disc tilt, 27 to 28 locations (33.75% to 35% of locations) showed differences greater than 3.9 μm across all VF-based paradigms, with differences reaching up to 18 μm, (Fig. 6, regression equations available in Supplementary Figure S4).

### Differences in measurements averaged over GII, GIII and GV

**Figure 7:**
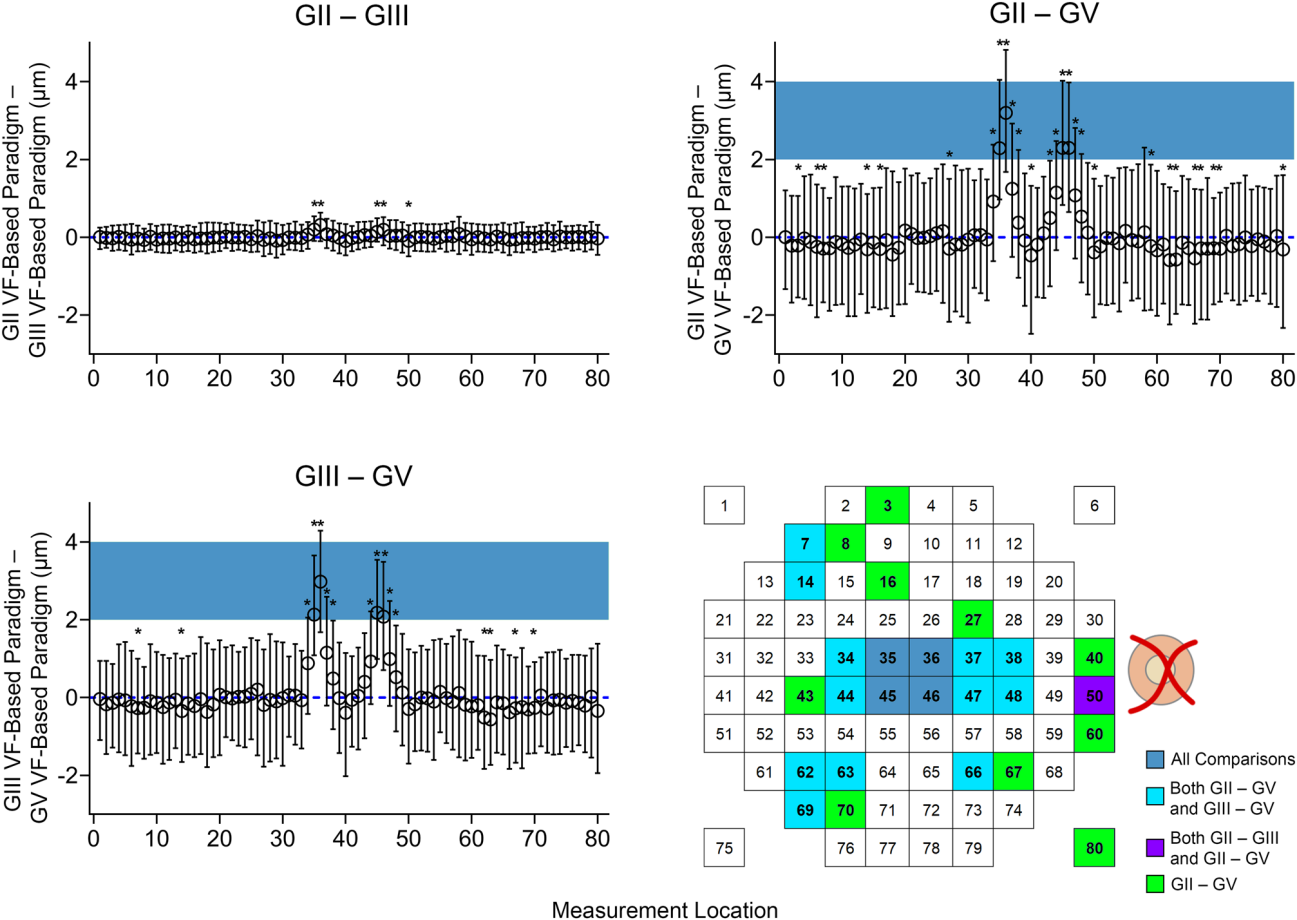
Scatter plots depicting differences in median ganglion cell-inner plexiform layer (GCIPL) thickness measurements when extracted using different visual field (VF)-based paradigms using Goldmann II, III and V (GII, GIII and GV); i.e. when measurements are averaged over different areas. Measurement locations were labelled as per the figure legend (bottom right). Individual data points (circles) indicate median differences between VF-based paradigms and standard measurements at each location, and error bars indicate interquartile ranges. The blue dashed line indicates no difference between the compared VF-based paradigms. Individual measurement locations showing the greatest deviations between the GV VF-based paradigm and the GII and GIII paradigms are highlighted in dark blue corresponding to foveal locations, as denoted in the figure legend. Locations marked with asterisks and in bold in the figure legend indicate significant deviations (Friedman test with multiple comparisons, P < 0.05). This figure was generated using Adobe Photoshop 2020 (Adobe Systems Incorporated, San Jose, CA, USA), with the scatter plots generated using GraphPad Prism Version 7.04 (GraphPad, La Jolla, CA, USA).

Furthermore, application of the custom algorithm enabled comparisons between GCIPL thickness measurements extracted over different VF-based paradigms, differing solely in areas over which measurements were extracted and averaged as per corresponding VF test sizes (Table 2, median GCIPL thicknesses available in Supplementary Figure S5). Overall, greater differences were observed between GII and GV VF-based paradigms and GIII and GV VF-based paradigms (Fig. 7), likely due to the similarity in area between GII and GIII. Interestingly, the interquartile ranges for these comparisons were larger than for GII and GIII VF-based paradigm comparisons, suggesting greater variation in GV GCIPL thickness measurements compared with GII and GIII. Across these comparisons, the greatest differences were observed in central foveal measurements, however these differences did not exceed 3.9 μm in any location.

## Discussion

The spatially targeted algorithm described in this study provides an accurate means of extracting GCIPL thickness values corresponding to projected inner retinal locations stimulated using standard central VF test patterns, as confirmed by algorithm validation. Furthermore, we demonstrate that GCIPL measurements extracted in this manner vary significantly from 8 × 8 grid measurements as extracted directly from the instrument review software, with greater differences observed when fovea to optic disc tilts deviate from 0. Our findings clearly indicate that the 8 × 8 grid inappropriately pools locations showing distinct variations in GCIPL thickness, despite its high sampling density (Fig. 4), and does not adequately represent the retinal locations sampled during VF testing, even in a normative cohort where, compared to a cohort with pathology, we would expect greater variability in GCIPL thickness values. Therefore, correlation of GCIPL measurements directly extracted from OCT review software with standard measures of visual function introduces a significant source of error in models of the macular structure–function relationship, with important implications for investigations of the glaucomatous structure–function relationship.

Previous studies have reported improvements in the observed macular structure–function relationship with precise matching of OCT-derived inner retinal measurements using customized software to the HFA 10-2 test grid^7, 16–18^. In particular, central test locations were notably impacted by correction for lateral displacement of the foveal GCs from their connecting photoreceptors^16,27^, which is largely consistent with our observations of the large foveal discrepancies between VF-based paradigms and the 8 × 8 grid; given that the VF-based paradigms indirectly exclude the foveal pit, the resultant GCIPL measurements more accurately reflect the high foveal GC density as reported in histological studies^6,13^. Additionally, studies using microperimetry, where VF test targets are projected directly onto the retina with correction for fixation losses via fundus tracking, have reported improved relationships between macular OCT measurements and VF results^35,36^, implying the importance of extracting structural and functional information from identical retinal locations. However, the approaches suggested by these studies have not been universally adopted in investigations of the macular structure–function relationship^9–12^, with several studies using OCT measurement paradigms averaging over larger areas than the 8 × 8 grid^9,11^, where the underlying region-specific variations in inner retinal thickness are captured less adequately. The potential impact of correlating non-corresponding measurements of structure and function across the macula has not been explored in detail previously.

The findings of the present study highlight the necessity of precise extraction of OCT measurements matching retinal locations stimulated with standard VF test grids. The utilized OCT measurement paradigms allowed for deviations in fixation due to microsaccadic eye movements, thereby partially compensating for the HFA’s disadvantage of lack of fundus tracking and resultant reduced certainty that structural measures topographically match those from retinal locations stimulated by VF test locations. Furthermore, as the OCT measurement paradigms match the HFA 10-2 and 30-2 test grids, our findings can be readily appropriated to clinical practice, where microperimeters are not routinely available.

An additional advantage of the described algorithm is its instrument-agnosticism with respect to the locations over which average OCT measurements are extracted. The HFA test grids were utilized due to the ubiquity of the HFA in clinical practice. Nevertheless, both locations and areas over which inner retinal measurements are averaged can be adjusted to match the test grid of the perimeter from which functional measurements are obtained. Moreover, customization of the locations from which OCT measurements are obtained is possible, which may be important to overcome individual anatomical variations in GC displacement at the macula. Turpin et al.^37^ reported that while the Drasdo model of GC displacement reasonably approximated the average GC displacement across their cohort, significant inter-individual differences in GC displacement remained, and the role of refractive error and axial length in contributing to these deviations is uncertain. Nevertheless, adjustments to test target location secondary to variations in GC displacement is possible with the present algorithm.

These approaches in optimizing image analysis methods do not only prompt the necessity of further work investigating the ideal parameters required to accurately describe the structure–function relationship in a research setting, but also have promising potential for clinical applications regardless of the VF instrumentation within a specific clinical setting.

Variations in position of the optic disc relative to the fovea have the potential to affect the location of projected VF test targets onto the retina, especially with atypical unusual optic disc positions or insertions^38^. In spite of this, there has been variation in how studies investigating the structure–function relationship have addressed this factor; some have aligned OCT measurement grids to individual fovea to optic disc tilts in order to minimize the introduced interindividual variation^7,20,25^, while others have not adjusted the tilt of the OCT measurement grid regardless of the underlying fovea to optic disc tilt^9,11,16^. Our observation that parafoveal locations approximately 6°–8° from the foveal center are most affected by fovea to optic disc tilt is in line with these locations demonstrating more notable variation in GCIPL thickness profiles across the macula, in contrast to peripheral macular locations that show relatively little variation (Fig. 4). These findings are largely consistent with previous histological and OCT-based studies investigating macular-wide variations in GC density and GCL thickness respectively^6,19,26^, indicating that the developed algorithm produces accurate measurements of the underlying GCIPL thickness when extracted as per the VF-based paradigms. Meanwhile, locations closer to the central fovea vary less due to smaller differences in location with the same difference in tilt, and therefore variations in fovea to optic disc tilts between individuals is less likely to impact these structural measurements.

Comparisons between different VF-based paradigms, differing solely by the stimulus size areas over which GCIPL measurements were averaged (Fig. 1), yielded the most notable differences at the central fovea, where GCIPL measurements averaged over the projected GV stimulus size were consistently reduced in comparisons with other VF-based paradigms, primarily due to encroachment of the larger stimulus size on the foveal pit. Nevertheless, differences between VF-based paradigms were consistently much less marked than in all comparisons with the 8 × 8 grid and did not exceed the axial resolution of the OCT, indicating that location of the extracted GCIPL measurements plays a more substantial role than area over which measurements are averaged. Perhaps of more interest is larger interquartile ranges in comparisons between the GV and other VF-based paradigms (Fig. 7), suggesting the presence of notable inter- and intraindividual variation in GCIPL thickness across the macula. While patterns of region-specific variation in the macular GCL have been described in detail^19,26^, current information on variations of the IPL and GCIPL within the normal macula relies heavily on OCT instrument-specific measurement grids^39–41^, and as such further work investigating normal variations in these layers with eccentricity using a greater sampling density is required. This in turn has important implications for future investigations of the relationship between macular GCIPL thickness and VF sensitivity.

## Limitations

A key limitation of the present study is that the applicability of OCT measurements extracted from the VF-based paradigms in accurate comparisons of structure and function relies on the assumption that participants are able to maintain steady central fixation on VF testing, which arguably applies to all investigations of the structure–function relationship using standard automated perimetry. We accounted for microsaccades by enlarging the areas over which the VF-based paradigms averaged OCT measurements, however this alone would not account for larger deviations in fixation that may occur during VF testing.

Only participants with spherical equivalent refractive error within ± 6.00 diopters and astigmatism < 3.00 diopters were included in the present study, as per previously reported exclusion criteria^19,26^. The validity of this algorithm on patients with high degrees of refractive error was not investigated in the present study, and it is possible that the projected target locations and areas may vary with higher degrees of refractive error or axial lengths. It may be worthwhile exploring the applicability of this algorithm on cohorts with high refractive error, to confirm its suitability for use in these cohorts.

## Conclusion

This study describes a novel and unique automated method to accurately extract OCT-derived GCIPL thickness measurements from locations and areas corresponding to projected VF test locations. We demonstrate that standard high-density OCT measurement paradigms, such as the 8 × 8 grid available on the Spectralis OCT review software, do not adequately reflect structural measurements corresponding to retinal locations sampled with standard VF test grids, particularly at central foveal locations where the greatest deviations were observed. Correction for retinal location and fovea to optic disc tilt are most pertinent in ensuring that precise GCIPL measurements reflecting visual function are obtained. By identifying and addressing a limiting factor in current investigations, the present study contributes a more complete understanding of the macular structure–function relationship and in turn aid development of future technologies that can detect early glaucoma with greater accuracy.

## Supplementary information

Supplementary Information
