# Supplementary material for: Custom extraction of macular ganglion cell-inner plexiform layer thickness more precisely co-localizes structural measurements with visual fields test grids
Source: Sci Rep. 2020 Oct 28;10:18527. doi: 10.1038/s41598-020-75599-0 (PMC7595126; doi:10.1038/s41598-020-75599-0)
Supplement: Supplementary file 1 — Supplementary Information [file 41598_2020_75599_MOESM1_ESM.pdf]

Supplementary Material for: Custom extraction of macular ganglion cell-inner plexiform layer thickness more precisely co-localizes structural measurements with visual fields test grids

Janelle Tong, BOptom,[1,2] David Alonso-Caneiro, PhD,[3] Nayuta Yoshioka, PhD, MOptom,[1,2] Michael Kalloniatis, PhD, MScOptom,[1,2] Barbara Zangerl, PhD, DVM[1,2]

1. Centre for Eye Health, University of New South Wales, Sydney, NSW Australia
2. School of Optometry and Vision Science, University of New South Wales, Sydney, NSW Australia
3. Contact Lens and Visual Optics Laboratory, Queensland University of Technology, Brisbane, QLD Australia

Number of figures: 5

Corresponding author:

Dr Barbara Zangerl

Centre for Eye Health, UNSW

Sydney 2052, NSW Australia

Email: bzangerl@cfeh.com.au

Phone: +61 2 8115 0793

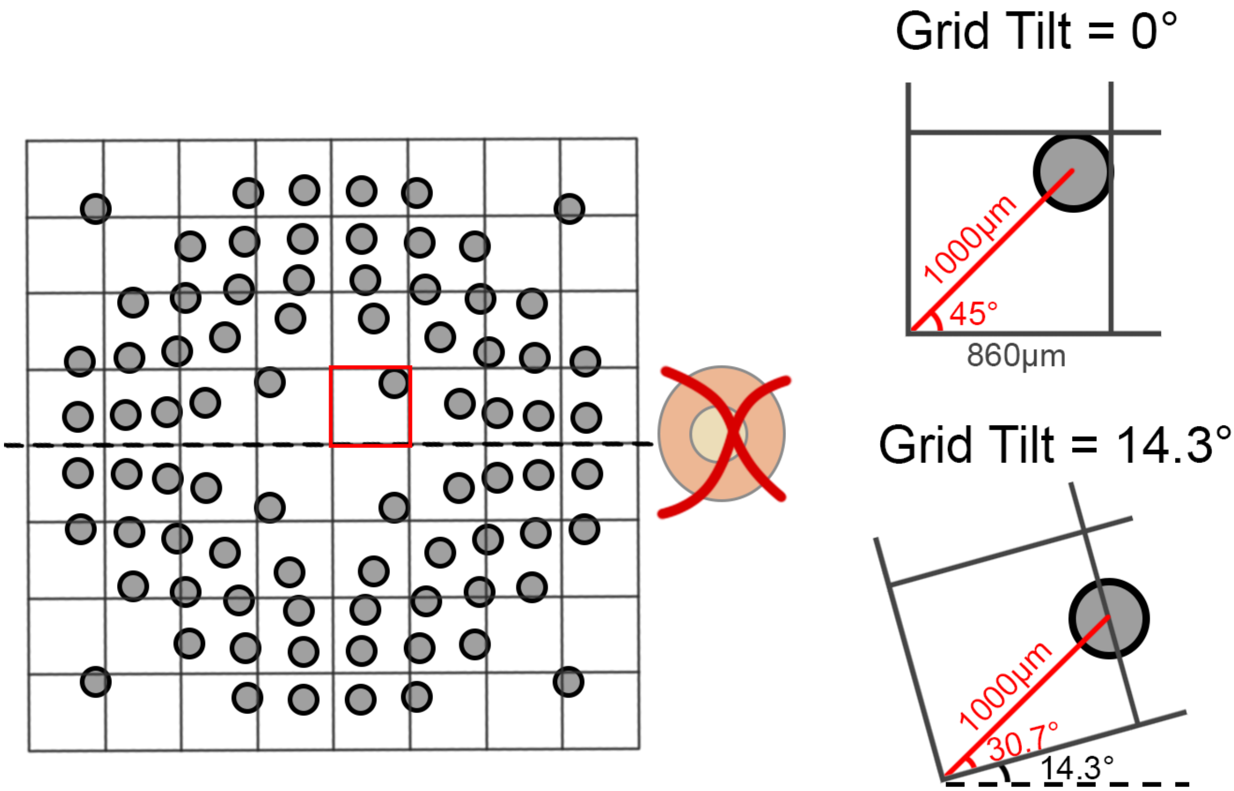

Supplementary Figure 1. Simplified schematic depicting how ganglion cell-inner plexiform layer (GCIPL) measurement comparisons between locations as per the visual field-based (VF-based) paradigm and as per the 8 x 8 grid were determined. For the VF-based paradigm location highlighted in red, the grid square with which it shared the greatest area was determined for a grid tilt of 0. Then, the threshold grid tilt in order for the VF-based paradigm measurement area to be shared equally by two adjacent grid squares was calculated using polar co-ordinates, such that for grid tilts greater than this threshold the VF-based paradigm location would be compared with the adjacent grid square. This process was repeated for negative grid tilts and for all other locations. This figure was generated using Adobe Photoshop 2020 (Adobe Systems Incorporated, San Jose, CA, USA).

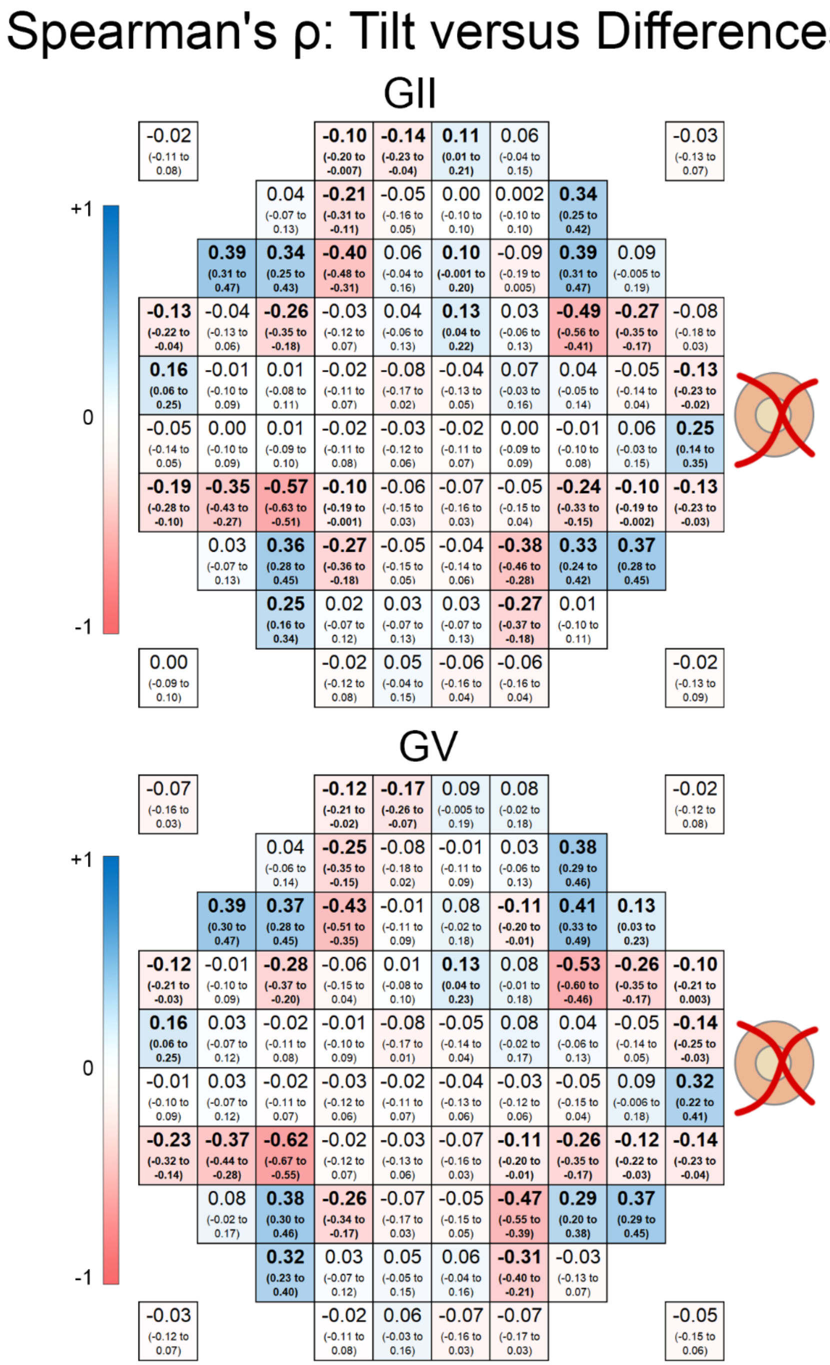

Supplementary Figure 2. Heat maps depicting location-specific variations in Spearman's rank correlation coefficients (ρ), calculated between optic disc to fovea tilts and differences in ganglion cell-inner plexiform layer (GCIPL) thickness between the Goldmann II (GII) and Goldmann V (GV) visual field (VF)-based paradigms and the 8 x 8 grid. Bold values indicate significant correlations (*P* <0.05) and values in brackets indicating the 95% confidence intervals of each Spearman's ρ. This figure was generated using Adobe Photoshop 2020 (Adobe Systems Incorporated, San Jose, CA, USA).

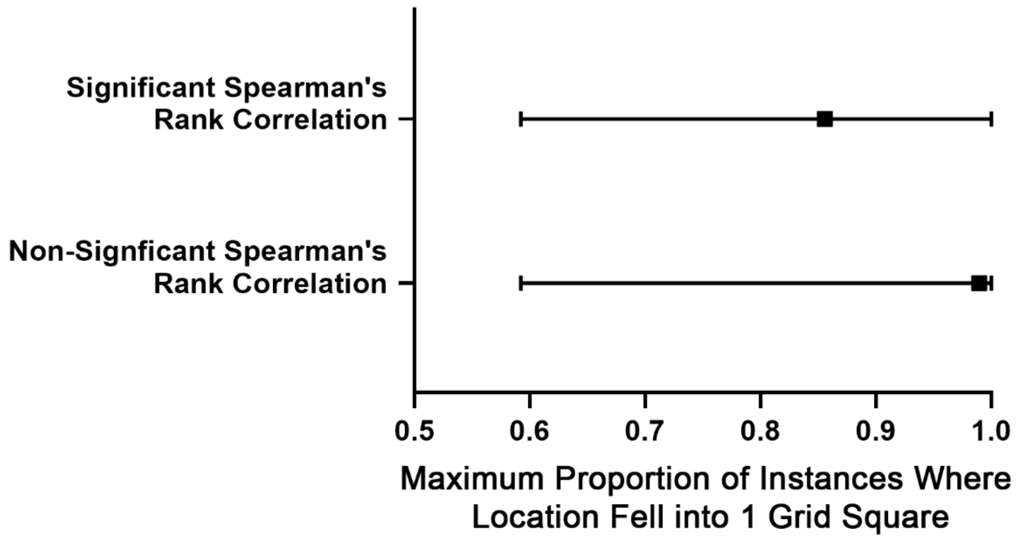

Supplementary Figure 3. For each location as per the visual field-based paradigms, across the study cohort the proportions of instances where locations fell on each grid square were calculated. Locations were categorized according to those that demonstrated significant Spearman's rank correlations between fovea to optic disc tilt and differences in GCIPL thickness between visual field-based paradigms and the 8 x 8 grid, and those that did not demonstrate significant Spearman's rank correlations. The maximum proportion was used as a surrogate measure of variability at each location; the closer the proportion is to 1, the more likely that the location consistently fell on the same grid square regardless of fovea to optic disc tilt. Locations demonstrating significant Spearman's rank correlations fell on different grid squares significantly more frequently (median 85.6% vs. 99.0%, $P < 0.0001$, Mann-Whitney test). Within the figure, the squares represent the median maximum proportion and the error bars represent the range of maximum proportions within groups. This figure was generated using GraphPad Prism Version 7.04 (GraphPad, La Jolla, CA, USA).

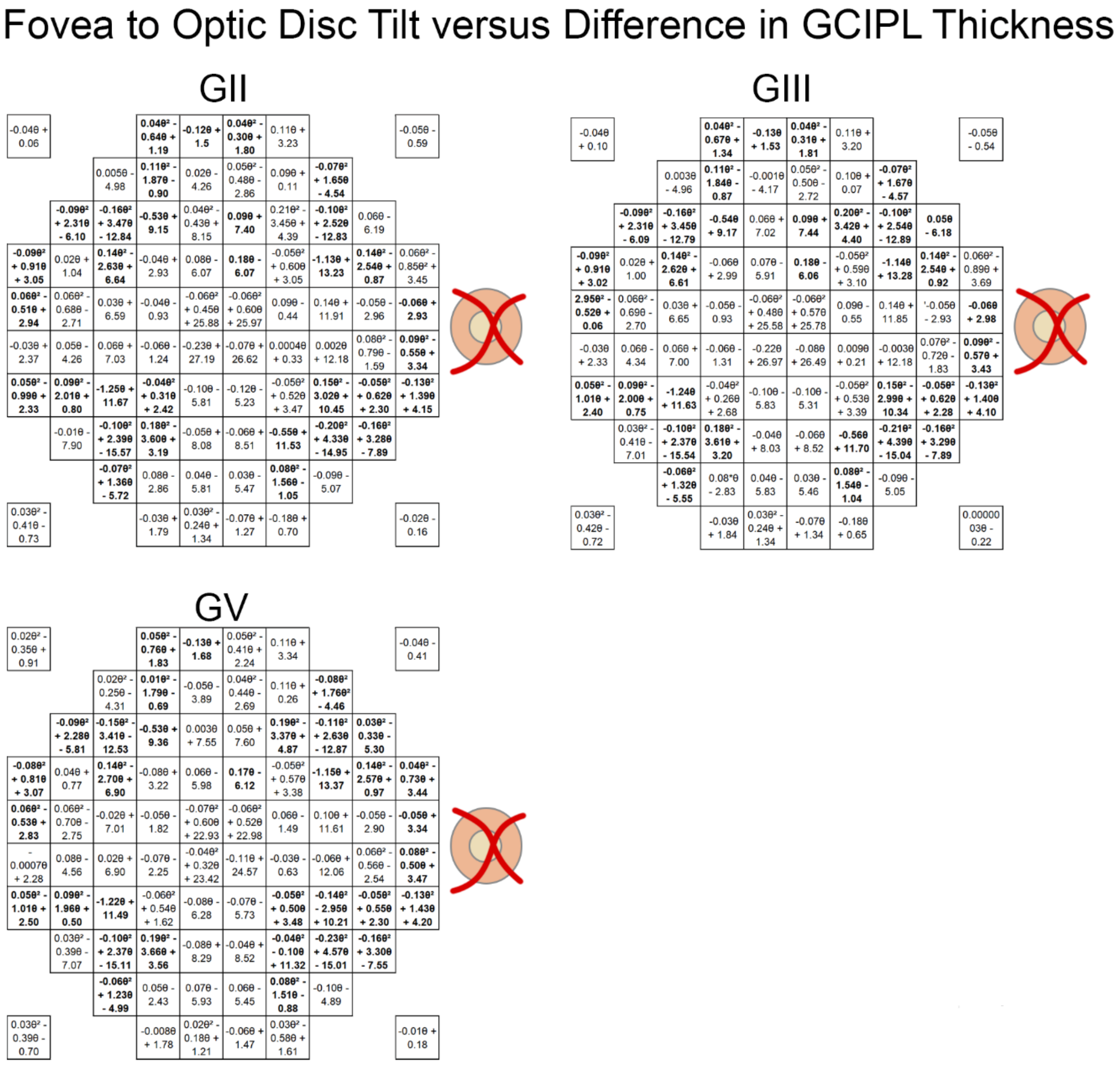

Supplementary Figure 4. Location-specific regression equations describing the relationship between fovea to optic disc tilt and differences in ganglion cell-inner plexiform layer (GCIPL) thickness between visual field (VF)-based paradigms and the 8 x 8 grid. The choice of quadratic or linear regression equation for each location and VF-based paradigm was determined using extra sum-of-squares F test, with the quadratic regression model chosen if the fit was significantly superior the linear regression model ($P < 0.05$). Θ indicates the fovea to optic disc tilt in degrees, and these equations were used to determine the difference values shown in Figure 6. Bold values indicate significant Spearman's rank correlation coefficients ($P < 0.05$) as per Figure 5 and Supplementary Figure 2. This figure was generated using Adobe Photoshop 2020 (Adobe Systems Incorporated, San Jose, CA, USA).

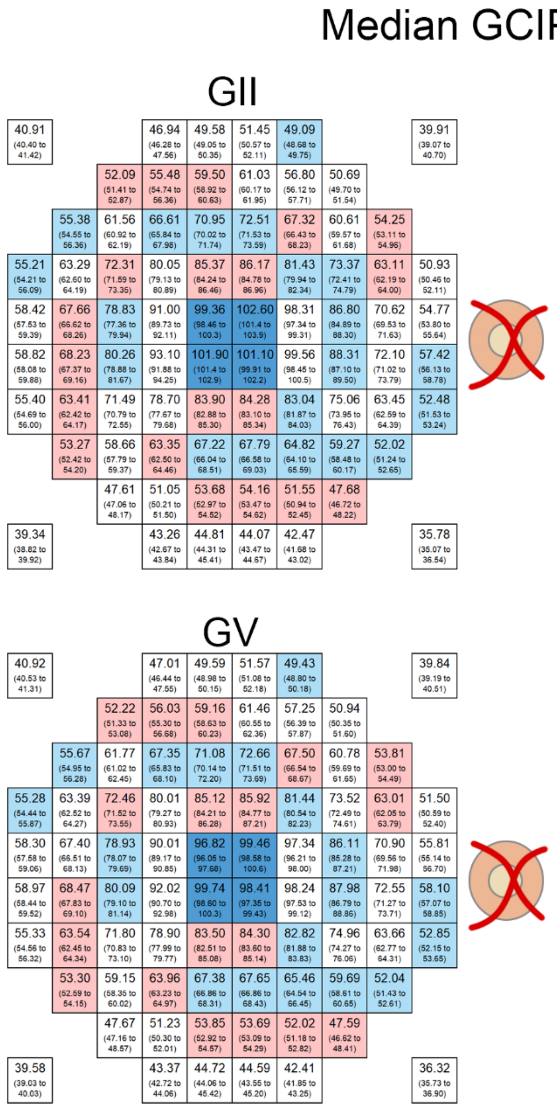

Supplementary Figure 5. Median ganglion cell-inner plexiform layer (GCIPL) thickness measurements as extracted by each visual field-based paradigm using projected Goldman II, III and V (GII, GIII and GV respectively) stimulus sizes. Values in brackets are the 95% confidence intervals for the median GCIPL measurement. As per Figure 3, measurement locations showing the greatest deviation are highlighted in dark blue, corresponding to foveal locations, and pale blue and pale red, corresponding to deviations greater than 3.9µm and less than -3.9µm respectively. This figure was generated using Adobe Photoshop 2020 (Adobe Systems Incorporated, San Jose, CA, USA).
